# Supplementary material for: Facile Synthesis of Mesoporous Silica at Room Temperature for CO2 Adsorption
Source: Micromachines (Basel). 2022 Jun 10;13(6):926. doi: 10.3390/mi13060926 (PMC9227262; doi:10.3390/mi13060926)
Supplement: Supplementary file 1 [file micromachines-13-00926-s001.zip › micromachines-1762184-supplementary.pdf]

Supplementary Information

Facile Synthesis of Mesoporous Silica at Room Temperature for CO_2 Adsorption

Misun Kang [1,2], Jong-tak Lee [2], Min Kyoung Kim [2], Myunghwan Byun [1,*], Jae Young Bae [2,*]

[1] Department of Advanced Materials Engineering, Keimyung University, Daegu 42601, Republic of Korea; misun.kang@gmail.com; myunghbyun@kmu.ac.kr
[2] Department of Chemistry, Keimyung University, Daegu 42601, Republic of Korea; misun.kang@gmail.com; jybae@kmu.ac.kr
* Correspondence: myunghbyun@kmu.ac.kr; jybae@kmu.ac.kr

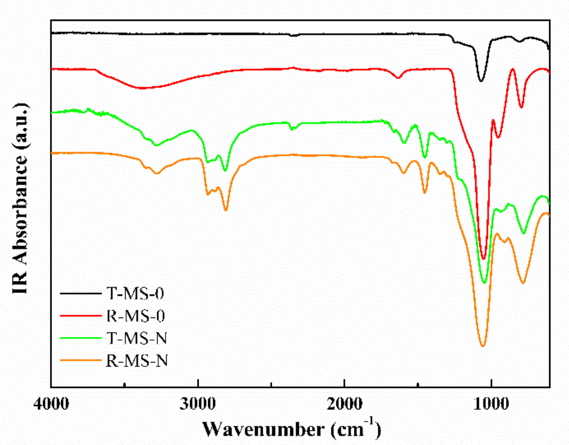

Figure S1. FT-IR spectra of silica materials with and without amine functional groups. The spectra of T-MS-0 and R-MS-0 is for confirming the surfactant removal process in the traditional method (black line) and in the room temperature process (red line), respectively. The other two spectra (green and orange) indicate that the mesoporous silica materials are loaded with amine functional groups.
